# Constipation

**Published:** 1859-12

**Authors:** 


### CONSTIPATION.

In the editor's book on Health and Disease—the third edition of which is called for within nine months after its first issue—is an article on this subject, which ought to be read and studied, and practically observed from early childhood by every human being. No person can be well long who does not have one action of the bowels every twenty-four hours, and to maintain a healthful regularity by means of natural agencies, such as by the ordinary food and drink is of the utmost importance to all. Some time in December, the third edition will be issued. Any of our subscriber sending to the editor one dollar, will receive it by mail, post-paid. But for those who may not feel inclined, or able to purchase the volume, we advise that whenever the bowels fail to act at the accustomed time, do not eat an atom of anything until they do act; in the meanwhile, drink as largely of cold water or herb tea as possible, keeping about in the open air, or follow steadily the ordinary avocations. It rarely happens that the fasting need be continued longer than twelve or fifteen hours; then eat moderately for a day or two; this is greatly better than the simplest medicine.

Any one having the tiniest mite of common sense, ought to know that if we continue to eat when there is no outlet, there must soon be a blow up in some direction. A lawyer in this city, a man of talent, energy and high character, assured us that until he saw our book, he imagined, and acted accordingly, that the best way to do "when constipated, was to eat more, and push it out." The result was frequent attacks of painful and dangerous disease.

